# Unraveling the Uncharacterized Domain of Carocin S2: A Ribonuclease *Pectobacterium carotovorum* subsp. *carotovorum* Bacteriocin

**DOI:** 10.3390/microorganisms10020359

**Published:** 2022-02-04

**Authors:** Ping-Chen Chung, Ruchi Briam James S. Lagitnay, Reymund C. Derilo, Jian-Li Wu, Yutin Chuang, Jia-De Lin, Duen-Yau Chuang

**Affiliations:** 1 Department of Anesthesia, Show Chwan Memorial Hospital, Changhua City 500, Taiwan; makotochung@gmail.com; 2 Department of Chemistry, National Chung-Hsing University, Taichung City 400, Taiwan; rslagitnay@nvsu.edu.ph (R.B.J.S.L.); rcderilo@nvsu.edu.ph (R.C.D.); janliwu2005@gmail.com (J.-L.W.); safata77429@gmail.com (J.-D.L.); 3 College of Arts and Science, Nueva Vizcaya State University-Bayombong Campus, Bayombong 3702, Nueva Vizcaya, Philippines; 4 College of Teacher Education, Nueva Vizcaya State University-Bambang Campus, Bayombong 3702, Nueva Vizcaya, Philippines; 5 Department of Entomology, National Chung-Hsing University, Taichung City 400, Taiwan; z789786@gmail.com

**Keywords:** antibacterial, bacteriocin, CaroS2K, CaroS2I, functional analysis, immunity protein, killer protein, *Pectobacterium carotovorum* subsp. *carotovorum*

## Abstract

Carocin S2 is a bacteriocin with a low molecular weight generated by *Pectobacterium carotovorum* subsp. *carotovorum* 3F3 strain. The caroS2K gene, which is found in the genomic DNA alongside the caroS2I gene, which codes for an immunity protein, encodes this bacteriocin. We explored the residues responsible for Carocin S2’s cytotoxic or RNA-se activity using a structure-based mutagenesis approach. The minimal antibiotic functional region starts at Lys691 and ends at Arg783, according to mutational research. Two residues in the identified region, Phe760 and Ser762, however, are unable to demonstrate this activity, suggesting that these sites may interact with another domain. Small modifications in the secondary structure of mutant caroS2K were revealed by circular dichroism (CD) spectroscopy and intrinsic tryptophan fluorescence (ITF), showing ribosomal RNA cleavage in the active site. A co-immunoprecipitation test indicated that the immunity protein CaroS2I binds to CaroS2K’s C-terminus, while a region under the uncharacterized Domain III inhibits association of N-terminally truncated CaroS2K from interacting with CaroS2I. Carocin S2, a ribosomal ribonuclease bacteriocin, is the first to be identified with a domain III that encodes the cytotoxic residues as well as the binding sites between its immunity and killer proteins.

## 1. Introduction

Bacteriocins as antibacterial agents have been the subject of extensive research in recent years. Bacteriocins are antimicrobial peptides produced by microorganisms that inhibit the growth of other microbes. Both Gram-positive and Gram-negative bacteria produce tiny ribosomally manufactured proteinaceous toxins that kill bacteria at precise concentrations [1,2,3,4]. Bacteriocins are antibacterial drugs with a restricted spectrum of activity that have biological activity against closely related bacterial species without disrupting commensal microflora [5]. Bacteriocin genetic material is commonly found on transposable elements, plasmids, or a producer’s chromosomes [6]. The creation of bacteriocin is triggered by stress signals and autolysis, and bacteriocin is liberated from the autolyzed cell after DNA damage triggers expression [7].

To avoid being killed by their own bacteriocins, bacteriocin-producing cells use something called immunity proteins, efflux pumps, or both. [8]. Bacteriocin enters a target cell by a particular receptor on its surface and kills it through a variety of processes, including ion-permeable channels in the cytoplasmic membrane, non-specific DNA degradation, protein synthesis suppression via targeted cleavage of 16S rRNA, and cell lysis [9,10].

There are four main types of bacteriocins generated by Gram-negative bacteria: colicins with a molecular weight larger than 10 kDa, which are produced by *E. coli*; a colicin-like protein with a molecular weight more than 10 kDa generated by bacteria other than *E. coli*., the microcin (with a molecular weight of less than 10 kDa), and the phage tail-like molecule (with a structure that is extremely similar to the phage tail) [11].

*Pectobacterium carotovorum* subsp. *carotovorum* (*Pcc*) is a phytopathogenic enterobacterium that causes soft-rot disease in a variety of plant species. Soft-rot pathogens have been found to inflict significant harm not just to vegetables but also to ornamental plants [12,13].

Despite the fact that *Pectobacterium carotovorum* species are phytopathogens that cause agricultural economic losses around the world, they produce one or more bacteriocins that let them compete with other related bacterial species [14]. Previous research demonstrated that *Pcc* produces low molecular weight bacteriocins such as carocin (S1, S2, S3, and D), pectocin (P, M1 and M2), and carotovorocin, a high molecular weight, phage-like bacteriocin with distinct properties [14,15,16,17,18]. Colicin-like bacteriocins (CLB) are the name given to these bacteriocins [19].

CLBs are multidomain proteins with domains for translocation, binding to receptors, and cytotoxicity. The N-terminal region of these proteins is intrinsically disordered, and it crosses the outer membrane to engage its translocation domains during translocation [20]. Endonuclease activity (DNAse, tRNAse, and rRNAse), depolarization of the inner membrane, and suppression of peptidoglycan synthesis are typically used by CLB generating strains to target a specific bacterial species through their cytotoxic domain [21,22]. We previously demonstrated that *Pcc*’s Carocins contain two proteins, one of which is responsible for antimicrobial activity (the killing protein) and the other for immunity (the immunity protein). These killer proteins have functional domains that include receptor binding, translocation, and DNase or RNase activity [17].

The receptor-binding domain of extracellular killer protein first detects and links the specific receptor on the membrane surface, then performs the mechanism of importation, and finally the translocation domain delivers the killer protein into the specified target within the vulnerable cell [23].

The C-terminal domain of the killer protein, in particular, affects the attacking mode of the killer protein once it enters the infected cell. Since the concurrent creation of the cognate immunity protein that normally interacts with the C-terminal domain of the killer protein, the bacteriocin producer is capable of particular immunity to the damage caused by its bacteriocin [24,25]. It’s worth noting that the immunity and killer proteins have a high affinity for each other due to charge attraction, and they’re separated at the cell surface by the energy generated by the proton motive force [26].

According to our previous findings, the carocin S2 gene is made up of two open reading frames: one with the 2352-bp *caroS2K* gene and the other with the 273-bp *caroS2I* gene. The ORF1 showed high homology to Carocin D, Colicin D, and Klebicin D, while the ORF2 showed homology to the immunity proteins of Colicin D and Klebicin D. The homology between the CaroS2K and Colicin D and Klebicin D is at the C-terminal end of these proteins, which is where the catalytic center of a ribonuclease is located. The amino acid sequence between Asp677 and the C-terminus of CaroS2K is over 60% comparable to the minimum tRNAse domain of Colicin D and Klebicin D, according to the researchers. Furthermore, the determined sequence of Carocin S2 was modeled using CLB’s Colicin D, Colicin E3, and Colicin E7 as a template. Carocin S2 may behave as a ribonuclease, according to our computer modeling, hence ribosomal RNA activity was examined.

*Pcc*’s Carocin S2 and its immunity protein were examined in vivo and in vitro in this study. Tests on the Carocin S2’s minimal domain suggest the region where the ribonuclease activity is located, implying the Carocin S2’s significant distinction from Colicin D, Klebicin D, and other CLB’s. Furthermore, the goal of this study was to establish an interaction site between caroS2I and the C-terminus of *caroS2K*.

## 2. Materials and Methods

*Bacterial strains, plasmids, media and growth conditions.* The bacterial strains, plasmids, and primers used in this study are listed in Table 1 and Table 2 and Supplementary Materials, Table S2. *Pcc* strains were grown at 28 °C in a modified Luria-Bertani (LB) medium containing 5 g of sodium chloride per liter (half from the recommended quantity of NaCl). *E. coli* strains (cloning host) were cultured at 37 °C with rotary agitation at 125 rpm in LB broth. 1% polypeptin, 0.2% yeast extract, 0.1% MgSO4 (pH 7.0), and 1.5% agar were added to the IFO-802 medium. Antibiotic concentrations used in *E. coli* selection: ampicillin; 50 g/mL; kanamycin, 50 g/mL; rifampicin, 50 g/mL were used to treat *E. coli* and *Pcc* strains. A spectrophotometer was used to detect all bacterial growth densities at 595 nm (OD_595_).

*Recombinant DNA techniques.* With the use of the various primers described in Supplementary Materials, Table S1, standard protocols for restriction endonuclease digestions, agarose gel electrophoresis, purification of DNA from agarose gels, DNA ligation, and other cloning related techniques were followed [27,28]. The protein-coding regions were identified using the BLAST software. MD Bio Inc. produced the oligonucleotide DNA primers (Taipei, Taiwan). For the PCR, Promega’s Go-Taq DNA polymerase was used. As previously mentioned, thermal asymmetric interlaced PCR (TAIL-PCR) was used [28,29].

*E. coli* DH5α cells that are exponentially growing (OD_595_ of about 1.0) were extracted for RNA extraction. RNA was extracted using Trizol reagent (Invitrogen, Waltham, MA, USA) and resuspended in DEPC-treated water according to the manufacturer’s instructions. The purity and concentration of total RNA were measured using a NanoVue PlusTM spectrophotometer (Biochrom US, Inc., Holliston, MA, USA), followed by electrophoresis on a 1.5% formaldehyde-morpholinepropanesulfonic-agarose gel for evaluation.

According to the manufacturer’s instructions, AMV Reverse Transcriptase (Promega, Madison, WI, USA) was used for Reverse Transcription-PCR (RT-PCR). For cDNA synthesis, one microgram (1 µg) of RNA was subjected to RT-PCR using CaroS2_re_1 (Supplementary Materials, Table S1) as a reverse primer.

Subcloning and transformation. With the primers CarocinS2K for2 and CarocinS2I rev2, the *caroS2K* gene was amplified from the genomic DNA of strain F-rif-18 and subcloned into pET32a to create the plasmid pEN2K. Excision of the tag element within the ribosome binding site and start codon of CaroS2K in pEN2K using the SLIM approach yielded the construct pEX2K, as previously described [30,31]. We used the primers 51HT32a4KI forT1, 51HTGT2KI forS, 51HT32a3KI revT, and 51HT32a4KI revS.

The pET30b-S2I (pEC2I) construct was isolated from an agarose gel and comprises a 273-bp amplicon carrying the *caroS2I* gene. It was digested with *NdeI* and *XhoI* and ligated onto pET30b that had been linearized using *NdeI* and *XhoI*. (Novagen). Similarly, the plasmid pES2I was created by excision of ORF2 from pEC2I with the (His)6-tag using the primers X2I_forT, X2I_forS, X2I_revT, and X2I_revS, as reported previously (Table 1). After then, the pEX2I construct was extracted from the kanamycin-resistant bacterial transformants, sequenced on both strands, and transformed into *E. coli* BL21 (DE3) cells (Novagen) [27,28].

*PCR site-directed ligase-independent mutagenesis.* Mutagenesis experiments were carried out using the plasmid construct pEN2K. Truncated CaroS2Ks expressed by the plasmids pES2TKD (400,450,600,677,691 and 692) were amplified using the primers specified in Table 2 and produced using the manner described previously [28].

*In vitro ribonuclease activity of Carocin S2.* In a dry bath incubator, a combination of one microgram (1 µg) total RNA isolated from indicator SP33, coupled with or without 1 µg purified CaroS2K protein in buffer A, was incubated at 28°C for 30 min. Formaldehyde gel loading buffer (0.2 g ethidium bromide, 10 mM EDTA (pH 8.0), 0.25% bromophenol blue, 0.25% xylene FF, and 50% glycerol) was used to mix the samples. The mixtures were electrophoresed on a 1.5% formaldehyde-MOPS agarose gel.

*Protein expression and purification.* In 500 mL LB medium, BL21 transformants harboring protein expression from the plasmids pEX2K or pEX2I were produced (OD_595_ 0.4). Isopropyl-D-thiogalactopyranoside (IPTG; final concentration, 0.1 mM; 25 °C for 12 h) was used to induce the cells. The cells were then pelleted, and the pellets were sonicated (10 cycles of 9 s with 9-s intervals). Ammonium sulfate precipitation (30–40%) was performed on BL21/pEX2K pellets, which were then resuspended in buffer a (30 mM NaCl and 20 mM Tris-Cl, pH 8.0) and applied to a Fractogel column (Merck, Kenilworth, NJ, USA). A NaCl gradient (30–1.4 mM) was used to elute the fraction. The CaroS2K fractions were pooled and concentrated using an Amicon centriprep-50 column (MilliporeSigma, Burlington, MA, USA) and dissolved in buffer A which contains 30 mM NaCl and 20 mM Tris-Cl, pH 8.0, after purification using a P-100 size-exclusion column (BioRad, Hercules, CA, USA). A similar chromatographic method employing the Amicon centriprep-3 column was used to purify CaroS2I. (MilliporeSigma, Burlington, MA, USA). Bradford assay was used to determine protein concentration (Amresco, Solon, OH, USA).

*Antiserum preparation for Carocin S2K and Carocin S2I.* By overnight culture in LB medium, BL21 was used to express carocinS2K and carocin S2I. The supernatants were centrifuged, then precipitated with 50% ethanol and loaded onto 10% SDS polyacrylamide gels. The proteins were electro-eluted after a direct cutoff of carocin S2K and carocin S2I was made. Rabbits were injected with the protein to create antibodies.

*Western blotting.* After electrophoretic transfer of proteins from an SDS-PAGE gel to a poly(vinylidene difluoride) (PVDF) membrane, western blot analysis was carried out. After electrophoresis, the proteins were electroblotted onto nitrocellulose in a semi-dry apparatus at 2 mA/cm^2^ for 20 min with a transfer buffer containing 40 40 mM glycine, 50 mM Tris, 0.4% SDS, and 10% methanol using a transfer buffer containing 40 mM glycine, 50 mM Tris, 0.4% SDS, and 10% methanol. After that, the nitrocellulose was saturated with gelatin, antibodies were added, and the blots were visualized using 3,3-diaminobenzidine (Sigma). Antibodies against CaroS2K or CaroS2I were diluted to 1:2000.

*Antibacterial activity assay.* Using the soft agar overlay method, the antibacterial activity of the bacteriocin producing strain was evaluated [28]. Overnight cultures of the indicator strain *Pcc* SP33 were inoculated in prewarmed LB with an optical density of 0.5 at 600 nm, then 100 µL was aliquoted into tubes with different Carocin concentrations. As for inhibitory assay, constant volumes of sodium phosphate buffer (30 mM NaCl, 20 mM NaH_2_PO_4_, pH 8.0 were prepared. Following that, the tubes containing the indicator strains and Carocin were incubated at 28 °C for one hour with shaking. The culture was plated on an LB plate and incubated for 16 h at 28 °C. The presence of growth-free inhibitory zones (clear zones) around the spotted area indicated the formation of an antibacterial substance [32].

*Determination of proteins secondary structure.* Circular dichroism (JASCO Model J-715, Tokyo, Japan) and Intrinsic Tryptophan Spectroscopy (JASCO FP-750, Tokyo, Japan) were used to predict the secondary structure of CarocinS2K and its related series of truncated bacteriocins [33,34,35]. Proteins were purified using medium pressure liquid chromatography prior to the experiment. The purified series of truncated Carocin S2K (10 µM) was eluted in a total volume of 200 µL with 20 mM Tris-HCl (pH 8.0), whereas the purified series of purified Carocin S2K (10 µM) was eluted in a total volume of 200 µL with NaH_2_PO_4_/Na_2_PO_4_ buffer. These solutions were loaded into a quartz cuvette and analyzed using far-UV CD with the spectral region ranging 160 to 240 nm [36] based on the manufacturers’ instructions. Near UV spectroscopy revealed a change in tryptophan in ITF (250–500 nm).

## 3. Results

### 3.1. Characterization of the Killer Protein (CaroS2K) Using Structure-Based Mutagenesis

The plasmid pMS2KI was used to clone the *carocin S2* gene sequence. Carocin S2’s killer protein was cloned into the plasmid pEN2K. Previous study suggested that a number of amino acids might be involved in the killer protein’s antibacterial activity. The CaroS2K sequence was utilized to generate a number of mutants with alanine substitutions, which were then compared in vivo to the wild type bacteriocin-producing strain.

**Figure 1:**
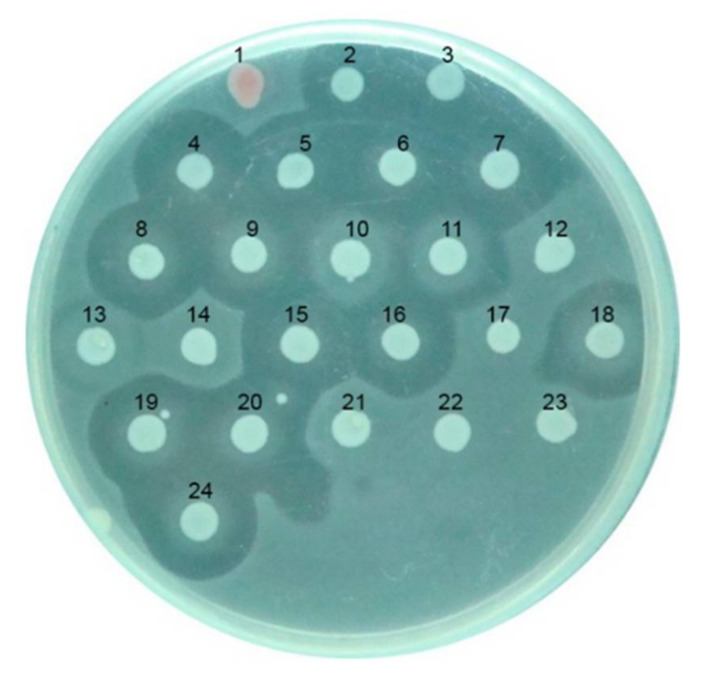
Mutation analysis of CaroS2K. (1) Serratia (indicator) (2) pEN2K (wild type) (3) R320A (4) K321A (5) T323A (6) K346A (7) T347A (8) T685A (9) K687A (10) K688A (11) K691A (12) K692A (13) K695A (14) H696A (15) K698A (16) S709A (17) Y734A (18) S740A (19) D755A (20) D757A (21) F760A (22) S762A (23) W764A (24) D767A.

The size of the inhibitory zone around each mutant was compared to the wild type (pEN2K) in a bacteriocin experiment. As shown in Figure 1, these findings point to a loss of antibacterial activity at various amino acid sites. These sites have amino acid sequences ranging from 691 to 764.

**Figure 2:**
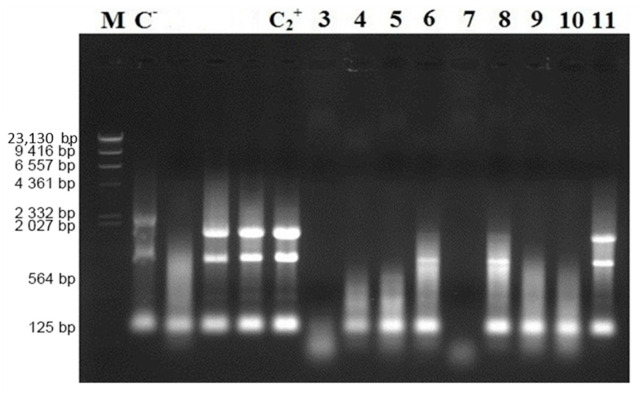
In vitro hydrolysis of RNA by CaroS2K and related mutants. Lane M, Lambda DNA- Hind III digested marker; Lane C-, negative control, SP33 *Pcc* strain total RNA (2µg); Lane C2+, SP33 positive control, SP33 *Pcc* strain total RNA (2µg) eluted in 10 µL Fractogel^®^ EMD TMAE (M) buffer; Lane 3, CaroS2K; Lane 4, K691A; Lane 5, K692A; Lane 6 K695A; Lane 7, H696A; Lane 8, Y734A; Lane 9, F760A; Lane 10, S762A; Lane 11, W764A.

An RNA degradation experiment was set up to confirm the RNA degradation activity of the various amino acid sequences of CaroS2K. In vitro evaluation of the RNAse activity of these amino acids revealed that both the wild type and mutant H696 hydrolyzed RNA (Figure 2). Surprisingly, mutant W764A lost entire activity, whereas the other mutant strains only showed partial hydrolysis.

**Figure 3:**
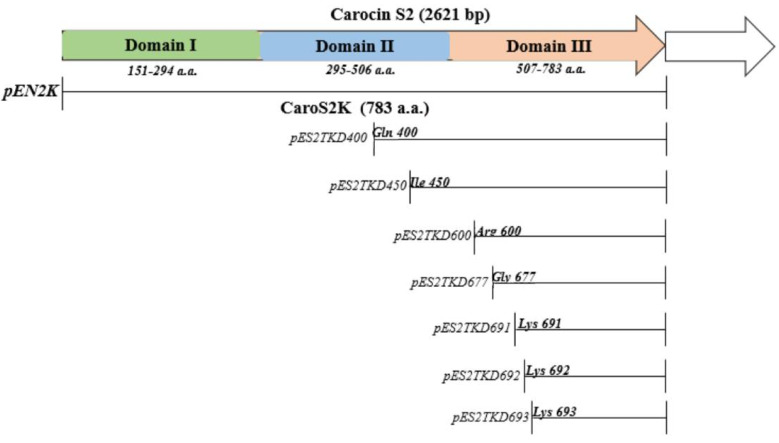
Physical map of Carocin S2 and mutants.

We generated shorter caroS2K mutants in an attempt to figure out where the cytotoxicity in the first set of site mutations lies.In the development of PEN2K segments, specific amino acid sequences (Gly 677, Gly 691, Lys 692, and Lys 693) as shown in Figure 3 were selected as a starting point for mutation.

### 3.2. Truncated Bacteriocin Isolation Expression, and Purification

**Figure 4:**
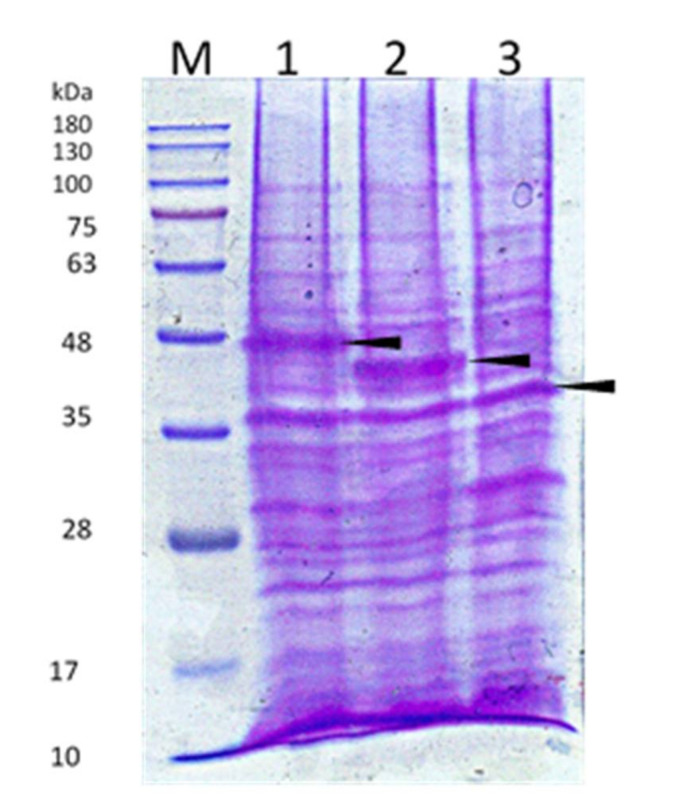
12% SDS-PAGE analysis of truncated bacteriocin producing mutant. Transform the pES2TKD series constructs that have been deleted into the protein expression host *E.coli* BL21, and add IPTG to 0.1mM to induce the production of bacteriocin protein. The arrow indicates the truncated bacteriocin expression band. (M) GeneDireX BlueRay Prestained Protein Ladder; (1) caroS2TKD400; (2) caroS2TKD450; (3) caroS2TKD600.

The truncated bacteriocin was expressed separately in *E. coli* BL21 (DE3) recombinants that had been transformed with pES2TKD series constructs. IPTG was used to induce all transformations. Purified truncated bacteriocin SDS-PAGE gels stained with Coomassie blue (shown in Figure 4) revealed a lane carrying the protein marker (Lane M), whose sizes (in kiloDalton) are indicated on the left. As demonstrated in Figure 4, all proteins expressed by the various mutants had the correct size and contained the CaroS2TKD mutation site inside the truncated bacteriocin protein, as shown by 12% SDS-PAGE.

According to earlier study, CaroS2K contains three putative domains and has been ruled out as a killer domain (extending from Asp677 to its carboxyl terminus). These constructs were used to better describe the Carocin S2 cytotoxic domain as well as to determine the minimal C terminal region.

### 3.3. Truncated Bacteriocin CaroS2TKD Mutants Were Tested for Ribosomal RNA Hydrolysis Activity

Since these shortened mutants are missing both the transmembrane and receptor binding functional regions of bacteriocin, they are likely to have already lost their antibacterial activity in vivo. The exact translation start of cytotoxic functional regions was investigated using in vitro Pcc ribosomal RNA hydrolysis assays..

**Figure 5:**
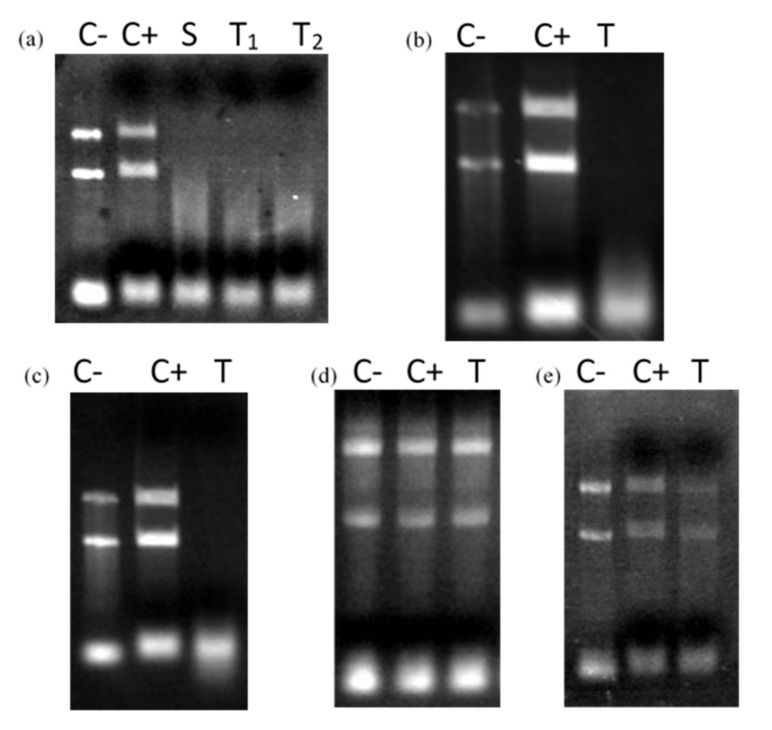
CaroS2K minimal ribonuclease region test. C−: *Pcc* strain SP33 total RNA 2 μg, which is negative control; C+: *Pcc* strain SP33 total RNA 2 μg, with Profinity IMAC resin extraction buffer to make up the reaction volume to 10 μL, which is positive control; S: caroS2K protein 400 ng, *Pcc* strain SP33 total RNA 2 μg, Profinity IMAC resin extraction buffer makes up the reaction volume to 10 μL; T: truncated bacteriocin caroS2TKD protein 400 ng, *Pcc* strain SP33 total RNA 2 μg, Profinity IMAC resin extraction buffer makes up the reaction Volume to 10 μL. Incubate for 3 h at 28 °C. (**a**) T1: caroS2TKD400, T2: caroS2TKD600; (**b**) caroS2TKD677; (**c**) caroS2TKD691; (**d**) caroS2TKD692; (**e**) caroS2TKD693.

Figure 5 illustrates that CaroS2TKD400, CaroS2TKD600, CaroS2TKD677, and CaroS2TKD691 still exhibit ribosomal RNA cleavage. RNA hydrolysis in vitro was still suppressed by a mutation at Lys692. This finding led to the conclusion that CaroS2K’s antibacterial activities are confined to Lys691 to Arg693.

**Figure 6:**
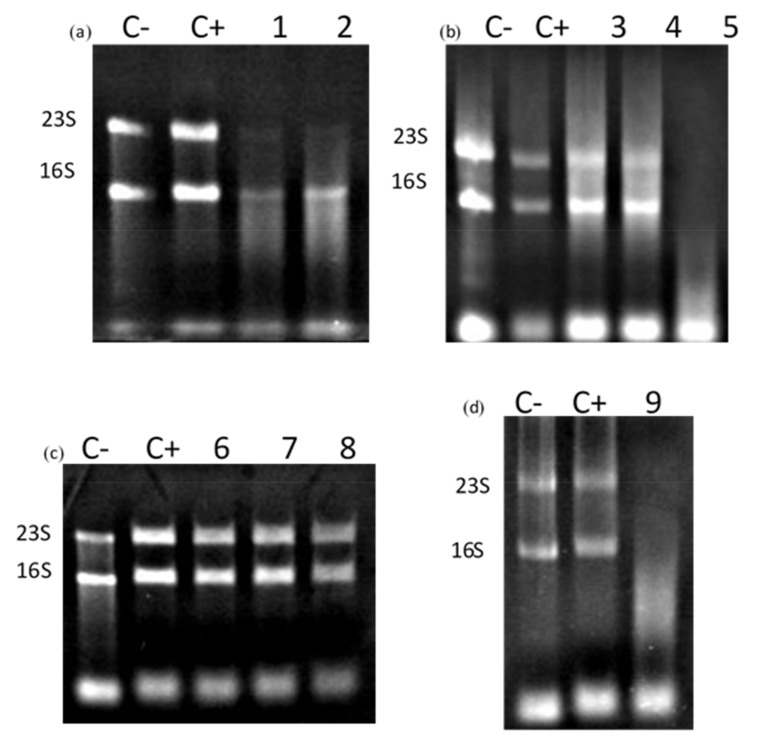
rRNA hydrolysis activity test of caroS2TKD677 mutants. C− *Pcc* strain SP33 total RNA 2 μg, which is a negative control; C+: *Pcc* strain SP33 total RNA 2 μg, with Profinity IMAC resin extraction buffer to make up the reaction volume to 10 μL, which is a positive control; (**a**) Inferred upstream area of the catalytic center, (1) Q688A; (2) K691A; (**b**) Predicted catalytic center, (3) K692A; (4) H696A; (5) Y734A; (**c**,**d**) Predicted downstream area of the catalytic center, (6) K695A; (7) F760A; (8) S762A; (9) W764A.

We created an alanine-substituted mutant construct derived from CaroS2TKD677 to examine the minimal C terminal region further. We identified changes in ribosomal RNA degradation activity after successfully altering nine mutants. With the exception of mutants Y734A and W764A, all mutants lost their ability to hydrolyze ribosomal RNA in vitro (Figure 6).This led us to an inference that the upstream catalytic center mutants are Q688A and K691A, the catalytic center mutants are K692A, H696A, and Y734A, while the downstream catalytic center mutants are K695A, F760A, S762A, and W764A.

### 3.4. Structural Depiction of Bacteriocin, Carocin S2

Using the CD spectrum, the structural differences between full-length Carocin S2 and related series of site-specific mutations were studied. The secondary structure of wild type is similar to that of the other three mutants in terms of structural folding (Supplementary Materials, Figure S1A). The looseness of the peaks was discovered when comparing wild type to all other mutants. The intrinsic tryptophan fluorescence (Supplemental Materials, Figure S1B) coincides with the CD result, although the structures generated by mutants K691A and K695A are tighter, as demonstrated by the Tryptophan fluorescence moving towards blue (Supplemental Materials, Figure S1B).

The secondary structure formed by the site-directed mutant strains and wild type is consistent in the configuration, according to the CD spectrum and ITF (Supplementary Materials, Figure S2A,B). Y734A and W764A mutations, on the other hand, result in blue shifts in proteins. Tyrosine is exposed to a higher extent than Tryptophan, indicating that the structure of protein is compact.

The CD map of mutant S762A (Supplementary Materials, Figure S3A) loosens by one unit when compared to the protein expressed by the wild-type strain. Furthermore, mutant S762A exhibits significant blue shifting of fluorescence when compared to the wild type (Supplementary Materials, Figure S3B).

**Figure 7:**
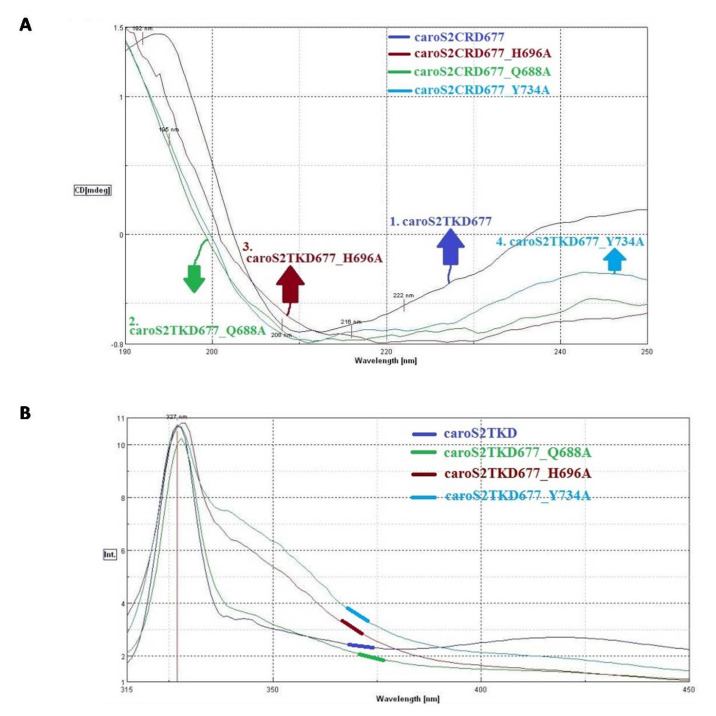
Carocin S2 structure analysis. (**A**) CD spectroscopy comparison chart of CaroS2−CRD677 and related series of mutants; (**B**) Comparison of ITF of CaroS2−CRD and related series of mutant strains.

As shown in Figure 7A,B, the sensitivity of the protein’s spectrum and fluorescence when altered from the minima area is higher than that of full-length mutants. Both investigations reveal that wild-type and site-specific mutant proteins have a looser shape than full-length proteins.

### 3.5. The Binding Region of CaroS2K and Its Immunity Protein CaroS2I

**Figure 8:**
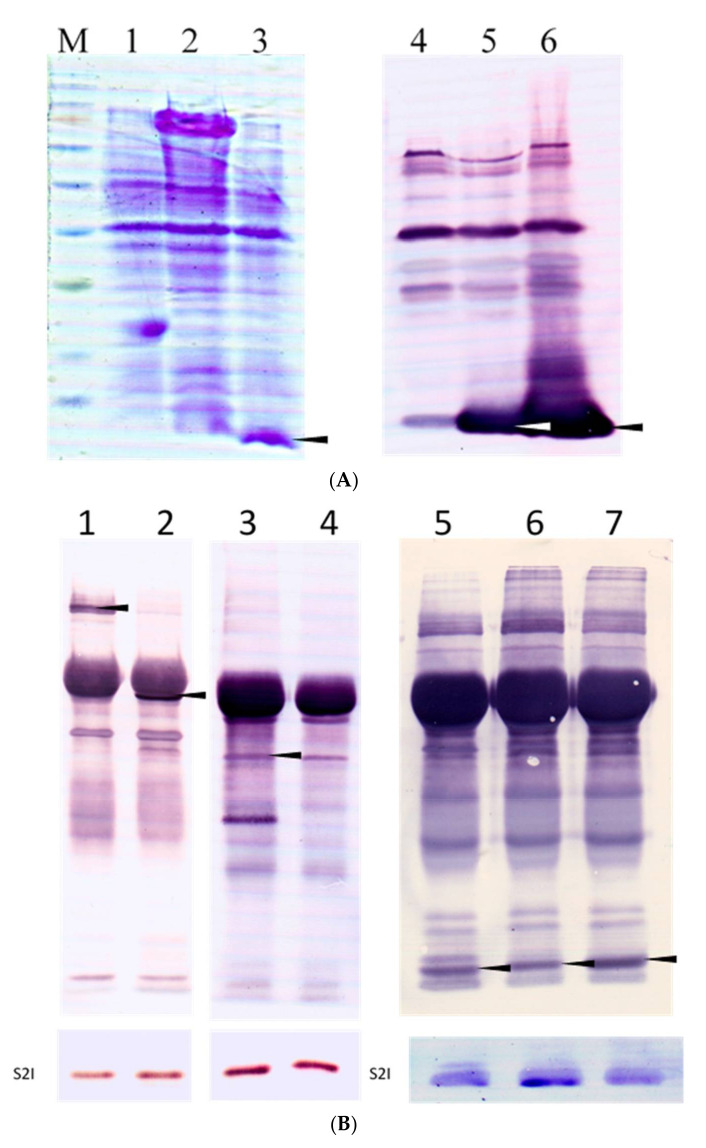
(**A**). Western blot analysis of the prepared caroS2I New Zealand white rabbit multi-strain antibody serum. The test construct was expressed by the host *E. coli* BL21 strain, the cell membrane was broken by heating after the induction of protein production, and directly analyzed by 12% SDS-PAGE and Western transfer method. The primary antibody is caroS2I multi-strain antibody serum, and the secondary antibody is Alkaline Phosphatase-conjugated Affinipure Goat Anti-Rabbit IgG. M: GeneDireX BlueRay Prestained Protein Ladder; (1,4) pET32a/BL21; (2,5) pES2KI/BL21; (3,6) pES2I/BL21. The arrow mark is the caroS2I protein expression band. (**B**). Western blot analysis of the co-immunoprecipitated truncated bacteriocins of different lengths. Each group uses caroS2K and caroS2I multi-strain antibody sera as primary detection antibodies. (1) caroS2K; (2) caroS2TKD400; (3) caroS2TKD600; (4) caroS2TKD677; (5) caroS2TKD691; (6) caroS2TKD692; (7) caroS2TKD693. The arrow mark is the truncated bacteriocin reaction color band of the experiment.

CaroS2I, an immunity protein, was expressed from the caroS2I gene with his-tag elements deleted at the 5′-end junction site. Multivalent antibody serum was generated from the isolated immunity protein. Western blot analysis was performed to establish the specificity of the antibodies generated after the 12% SDS-PAGE of CaroS2I protein (Figure 8A).

A co-immunoprecipitation test was used to look at the binding sites of the bacteriocin killer protein CaroS2K and the immunity protein CaroS2I. Full-length CaroS2K bacteriocins, as well as truncated bacteriocins of various lengths, were studied in this work. The findings revealed bands in the molecular weight area of the caroS2TKD protein, indicating that caroS2I binds to the C-terminal cytotoxic functional portion of caroS2K. (Figure 8B).

## 4. Discussion

Based on the amino acid sequence inferred from the nucleotide sequence of the *carocin S2* gene using DNASIS-Max software version 3.0 (Hitachi Software Engineering Co., Ltd., Tokyo, Japan), CaroS2K was found to be highly conserved among the various strains (Supplementary Materials, Figure S4). Variations in nucleotide sequences demonstrated good conservation at the C-terminal amino acid level during a multiple sequence alignment of CaroS2K to other bacteriocins (UniprotKB F8RN7, Uni-protKB P17998, UniprotKB Q5QGN1, UniprotKB A6V5R2, UniprotKB Q06584).

According to computational techniques using multiple bioinformatic tools applied to the *carocin S2* gene sequence, the functional region of the CaroS2K killer protein is organized, and this is located in the protein’s C-terminal killing domain. A region of about 220 amino acids in the receptor-binding domain is dubbed Domain III for the time being, but its function is still uncertain.

A killer gene, which encodes a killer protein that limits the growth of sensitive cells, and an immunity gene, which encodes a protein that confers particular immunity against the killer protein, make up the bacteriocin operon [4,5].

Domain III, which has yet to be uncovered, is produced by CLB crystals. This is the first study to look into Carocin S2′s killing domain, which was translated starting at residue Ile450, which is positioned at the intersection of the receptor-binding domain and Domain III, while keeping the receptor-binding domain’s downstream sequence. Carocin S2 producing strains and related site-directed mutants, as well as N-terminally truncated Carocin S2 producing strains and related site-directed mutants, were investigated in vivo and in vitro.

The alanine-scanning mutagenesis indicated that several amino acid residues were required for CaroS2K’s cytotoxicity. 

The 60% homology of CaroS2K to Colicin D explains some structural and topological similarities. Colicin’s inability to demonstrate the zone of inhibition [37,38] corresponds to CaroS2K Lysine mutations. In contrast, the CD and ITF spectra revealed that the Lysine residue has been inverted, showing the Tryptophan in the structure. The active site catalyzes the hydrolysis of RNA, causing the Lysine residue to play a significant role in substrate binding, according to the flipping effect. The substrate rRNA backbone’s charged phosphate groups can be neutralized by lysine residues, which form a positive charge cluster.

The in vivo activity of the histidine residue mutant against the indicator strain was reduced, but it was still capable of completely hydrolyzing RNA. This discovery is attributed to Domains I and II, and it contradicts the hypothesis that His 696 is the protein’s catalytic site, but it could still be a structural core. It was discovered that hydrophobic residues have a similar impact.

Furthermore, the rRNA binding residue of CaroS2K was compared to Colicin according to a study on the Ser677 mutation in Colicin [39], it may play a function in substrate binding and catalysis. The mutant has been discovered to interact directly with the RNA substrate and is essential for RNA binding. On the other hand, our data demonstrated that mutant Ser762 still has some extracellular cytotoxicity. The discrepancy with the expected outcome can be traced to a slight difference in the amino acid structure since the observed secondary structure is loose compared to the wild type. Colicin D, on the other hand, does not have this property, implying that it interacts with other domains.

The interaction region is designed to resemble the molecular backbone of rRNA, which is protected by a pseudo-substrate. CaroS2I binds to the cytotoxic portion of CaroS2K within the protein’s Domain III, as evidenced in a series of truncated bacteriocins created during the attempt to minimize the cytotoxic section of CaroS2K. The fact that the CaroS2TKD691, CaroS2TKD692, and CaroS2TKD693 N-terminally deleted proteins bind to CaroS2I shows that the killer-immunity protein interaction area is downstream of His696.

According to the findings of the study, the CaroS2TKD677 with the conserved Domain III sequence demonstrated a unique resistance binding action. Since mutants with longer amino acid sequences (CaroS2TKD400 and CaroS2TKD600) do not impact the cytotoxic domain’s interaction with caroS2I, the length of the Domain III sequence is assumed to vary the folding configuration of this area. This shows that the extended sequence of short fragments may change the structure of proteins and impede immune protein binding. Carocin S2 is an RNAse that limits protein synthesis in the cytoplasm by degrading either 23S or 16S rRNA. CaroS2I is an immunity protein that prevents CaroS2K from harming cells in the cytoplasm [16].

### Proposed Catalytic Mechanism

The general catalytic mechanism by which ribonucleases destroyed RNA has been studied for decades using protein engineering and crystallographic analysis. RNase A, for example, has two active sites, His residues, that cooperate during the catalytic cycle. One acts as a general base, attracting a proton from the ribose 2′-OH and catalyzing the nucleophilic attack of this radical on the 3′-phosphate group, resulting in a cyclic intermediate, while the other acts as a catalytic acid during the initial cyclization step. Their catalytic roles are reversed during the subsequent hydrolysis of the cyclic intermediate. Barnase and colicin E3 are ribonucleases that act in a similar way, but with a His and a Glu as catalytic residues [39].

The probable active site region of CaroS2K was investigated by systematic SLIM of residues on the catalytic surface (Supplementary Materials, Figure S5). The loss of catalytic activity when this His is replaced with Ala supports the hypothesized structure that His696 is the general foundation of the reaction’s cyclization step. The ribose 2′-OH nucleophilic attack on the 3′-phosphate group results in the development of a charged penta-covalent transition state that can be stabilized by surrounding charged residues. Lys692 in CaroS2K has amine groups that are well-positioned to execute this function. Their mutation to Ala abolishes RNase activity but not CaroS2K cytotoxicity, implying that they are involved in RNA substrate recognition or, more specifically, catalytic activitySer762 is necessary for both cytotoxic and RNase activity, showing that Ser762 is involved in substrate binding and/or catalysis. Near the His696 imidazole side chain, Tyr734, Phe760, and Trp764 form a hydrophobic platform, exposing one face of its indole ring to the solvent and stacking on an RNA base. The lack of activity in the Tyr734Ala, Phe760Ala, and Trp764Ala mutants supports this hypothesis. 

Using site-directed mutagenesis, we were able to map the CaroS2K active site and identify residues that are critical for cytotoxicity (Lys692, His696, Tyr734, Phe760, Ser762 and Trp764). Two important domain III residues, Phe760 and Ser762, have been found to interact with another domain. As a result, it is the first publication on the investigation of the uncharacterized domain III of Carocin S2, a Colicin-like bacteriocin.

## 5. Conclusions

Carocin S2, a Colicin-like bacteriocin generated by *Pectobacterium carotovorum* subsp. *carotovorum*. Carocin S2 encodes a killer protein in Domain III and an immunity protein in the C-terminal of the killer protein’s cytotoxic functional domain. The protein’s cytotoxicity and RNA cleavage activities were shown to be linked to eight residues. Phe760 and Ser762 were found to interact with another domain. These findings will contribute to our understanding of Carocin S2, a novel bacteriocin.

## Figures and Tables

**Table 1:** Bacteria and Plasmids used in this study.

Strain or Plasmid	Relevant Characteristics	Source of Reference
*Pectobacterium carotovorum* subsp. *carotovorum*		
F-rif-18	*Pcc*, Rif^r^, wild-type	Laboratory stock
SP33	Wild type, indicator	Laboratory stock
*Escherichia coli*		
DH5α	supE44ΔlacU169(ψ80 lacZΔM15)hsdR17 recA1 endA1 gyrA96 thi-1 relA1	Laboratory stock
BL21 (DE3)	hsdS gal(λ*c*I*ts*857 *ind*1 Sam*7 nin5* l*ac*UV5-T7 gene 1)	Laboratory stock
Plasmid		
pGEM T-Easy	Amp^r^; lacZ cloning vector	Promega
pET30b	Kan^r^; expression vector with the C-terminal His-tag	Novagen
pET32a	Amp^r^; expression vector with the N-terminal His-tag	Novagen
pEN2K	*caroS2K* subcloned into pET32a	This study
pEX2K	Derived from pEN2K; deleted series of Tag-element in front of expressed *caroS2K*	This study
pEC2I	Caros2I subcloned intro pET30b	This study
pEX2I	Derived from pEC2I; deleted series of Tag-element in front of expressed *caroS2I*	This study
pEH2K	Derived from pEC2I; adding (His)_6_-Tag adjacent to *caroS2I*	This study
pES2TKD400	N-terminally truncated Carocin S2 at Gln400	This study
pES2TKD600	N-terminally truncated Carocin S2 at Arg600	This study
pES2TKD677	N-terminally truncated Carocin S2 at deleted Gly677	This study
pES2TKD691	N-terminally truncated Carocin S2 at deleted Lys691	This study
pES2TKD692	N-terminally truncated Carocin S2 at deleted Lys692	This study
pES2TKD693	N-terminally truncated Carocin S2 at deleted Lys693	This study

Rif^r^, -Rifampicin resistance

**Table 2:** Bacteria and Plasmids used in this study.

Primers	Sequence (5′-3′)
CaroS2I_C-taq_for	CATATGATGAGTAATAAACT
CaroS2I_C-taq_rev	CTCGAGAAGAAGTTTGAA
X2I_forT	GGAAAAATTCAAACTTCTTTGAGATCCGGCTGCT
X2I_forS	TGAGATCCGGCTGCT
X2I_revT	AAGAAGTTTGAATTTTTCCAACGTGGCTTTTATTTC
X2I_revS	AACGTGGCTTTTATTTC
CarocinS2K_for2	CGGTCAGGATCCATGATTAAGTAC
CarocinS2I_rev2	GCGCCAAAGCTTCAAGAGATATCA
5IHT32a2KI_forT	GAAGGAGATATACATATGATTAAGTACCGTTTATA
5IHTGT2KI_forS	ATGATTAAGTACCGTTTATA
5IHT32a3KI_revT	ATGTATATCTCCTTCTTAAAGTTAAACAAAATTATTTC
5IHT32a4KI_revS	TTAAAGTTAAACAAAATTATTTC
C2KIH_forT	CACCACCACCACCACCACTGATATCTCAAGCTTGCG
C2KIH_forS	TGATATCTCAAGCTTGCG
C2KIH_revT	GTGGTGGTGGTGGTGGTGAAGAAGTTTGAATTTTTCC
C2KIH_revS	AAGAAGTTTGAATTTTTCC
S2TKD667_revT	CATATGTATATCTCCTTCTTAAAGTTAAACAAAATTATTTC
5IHT32a4KI_revS	TTAAAGTTAAACAAAATTATTTC
S2TKD400_forT	GAAGGAGATATACATATGCAGGCTTATTTCAGAGC
S2TKD400_forS	CAGGCTTATTTCAGAGC
S2TKD450_forT	GAAGGAGATATACATATGATAAAACGCAACAGGGT
S2TKD450_forS	ATAAAACGCAACAGGGT
S2TKD500_forT	GAAGGAGATATACATATGAAGTCACAAGGGATGATTGG
S2TKD500_forS	AAGTCACAAGGGATGATTGG
S2TKD600_forT	GAAGGAGATATACATATGCGCCTTGTACTGGAAAACC
S2TKD600_forS	CGCCTTGTACTGGAAAACC
S2TKD677_forT	GAAGGAGATATACATATGGATCCCTTGGATTCAGATCGG
S2TKD677_forS	GATCCCTTGGATTCAGATCGG
S2TKD691_forT	GAAGGAGATATACATATGAAAAAGTATCTTAAACATGCC
S2TKD691_forT	AAAAAGTATCTTAAACATGCC
S2TKD692_forT	GAAGGAGATATACATATGAAGTATCTTAAACATGCC
S2TKD692_forS	AAGTATCTTAAACATGCC
S2TKD693_forT	GAAGGAGATATACATATGTATCTTAAACATGCCAAAG
S2TKD693_forS	TATCTTAAACATGCCAAAG

## Data Availability

The datasets used and analyzed during the current study are available from the corresponding author on reasonable request.

## Supplementary Materials

The following supporting information can be downloaded at: www.mdpi.com/article/10.3390/microorganisms10020359/s1, Table S1. Plasmids used in this study; Table S2. Additional primers used in this study; Figure S1. Structure analysis of the CaroS2K and its related mutants; Figure S2. Structure analysis of the CaroS2K and its related mutants; Figure S3. Structure analysis of the CaroS2K and its related mutants; Figure S4. The multiple sequence alignment of CaroS2K with other bacteriocins; Figure S5. Proposed hydrolysis mechanism of CaroS2K
