# Supplementary material for: Unraveling the Uncharacterized Domain of Carocin S2: A Ribonuclease Pectobacterium carotovorum subsp. carotovorum Bacteriocin
Source: Microorganisms. 2022 Feb 4;10(2):359. doi: 10.3390/microorganisms10020359 (PMC8878655; doi:10.3390/microorganisms10020359)
Supplement: Supplementary file 1 [file microorganisms-10-00359-s001.zip › microorganisms-1535649-supplementary.pdf]

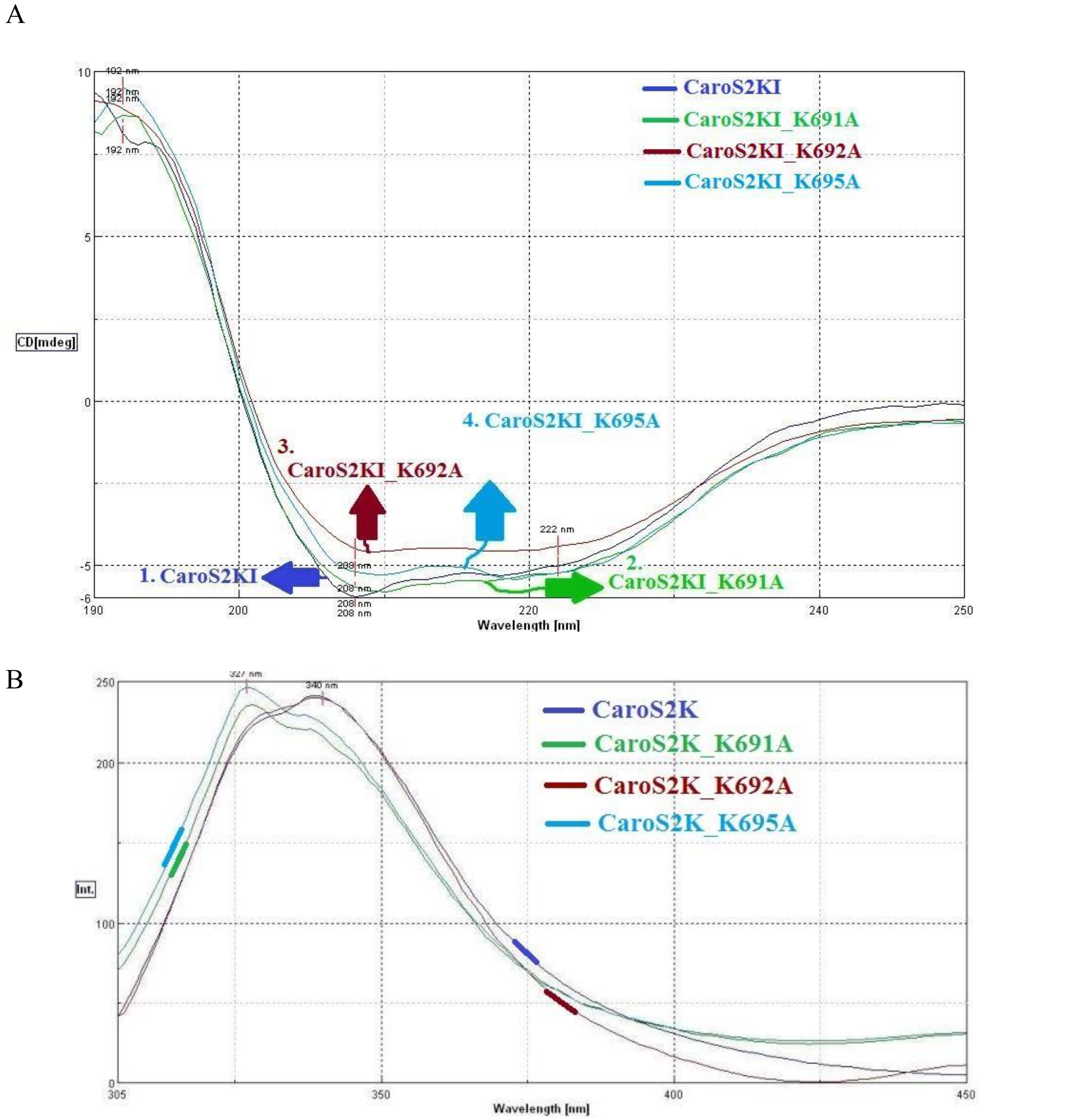

Supplementary material, Figure S1. Structure analysis of the CaroS2K and its related mutants. (A) CD spectroscopy comparison chart of CaroS2K and Lysine residue mutants (B) ITF comparison of CaroS2K with Lysine residues.

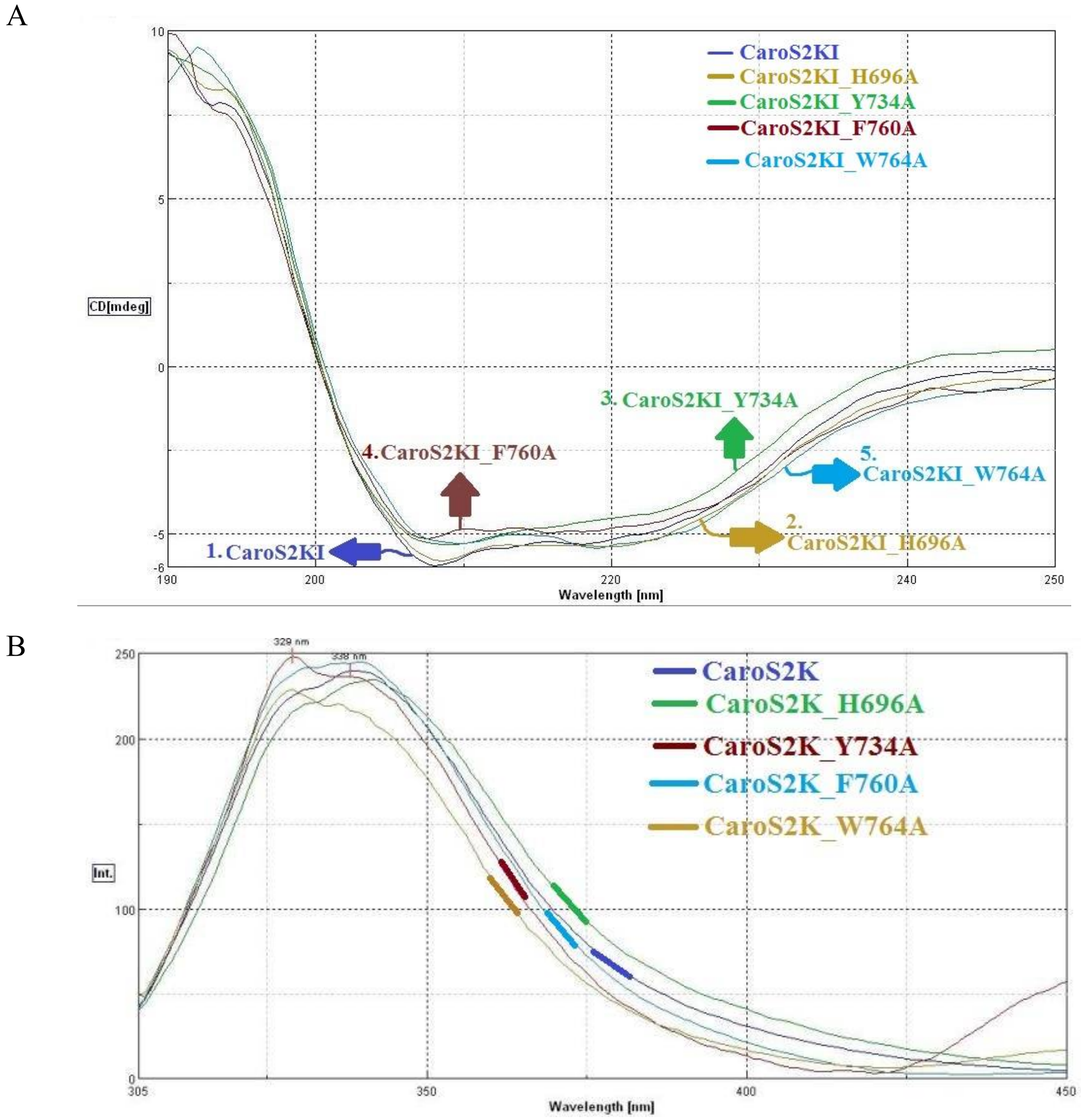

Supplementary material, Figure S2. Structure analysis of the CaroS2K and its related mutants. (A) CaroS2K, the Histidine residue mutant, and hydrophobic residue mutants CD spectroscopic comparison chart (B) ITF comparison of CaroS2K, the Histidine residue mutant, and hydrophobic residue mutants

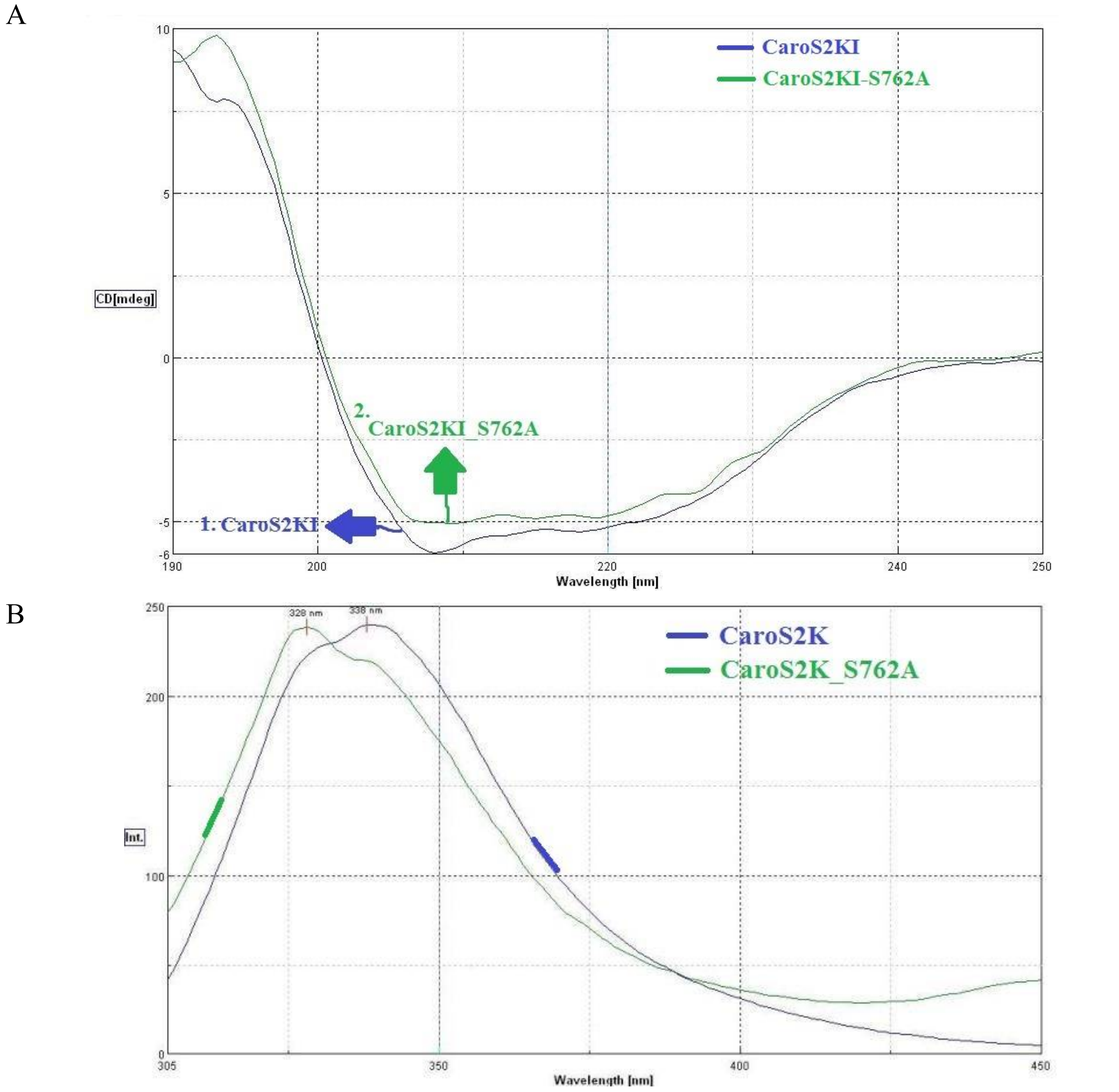

Supplementary material, Figure S3. Structure analysis of the CaroS2K and its related mutants. (A) CaroS2K and RNA binding residue mutant CD spectroscopic comparison chart (B) CaroS2K and RNA binding residue mutant ITF comparison.

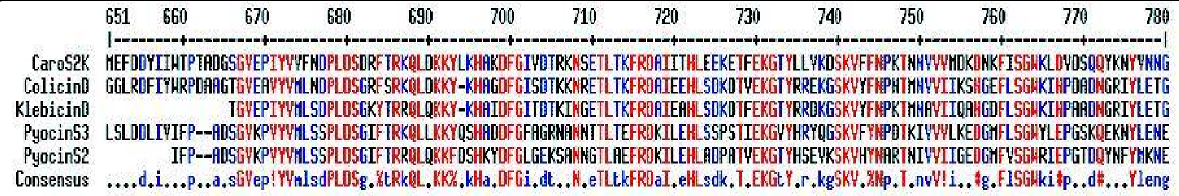

Supplementary material, Figure S4. The multiple sequence alignment of CaroS2K with other bacteriocins.

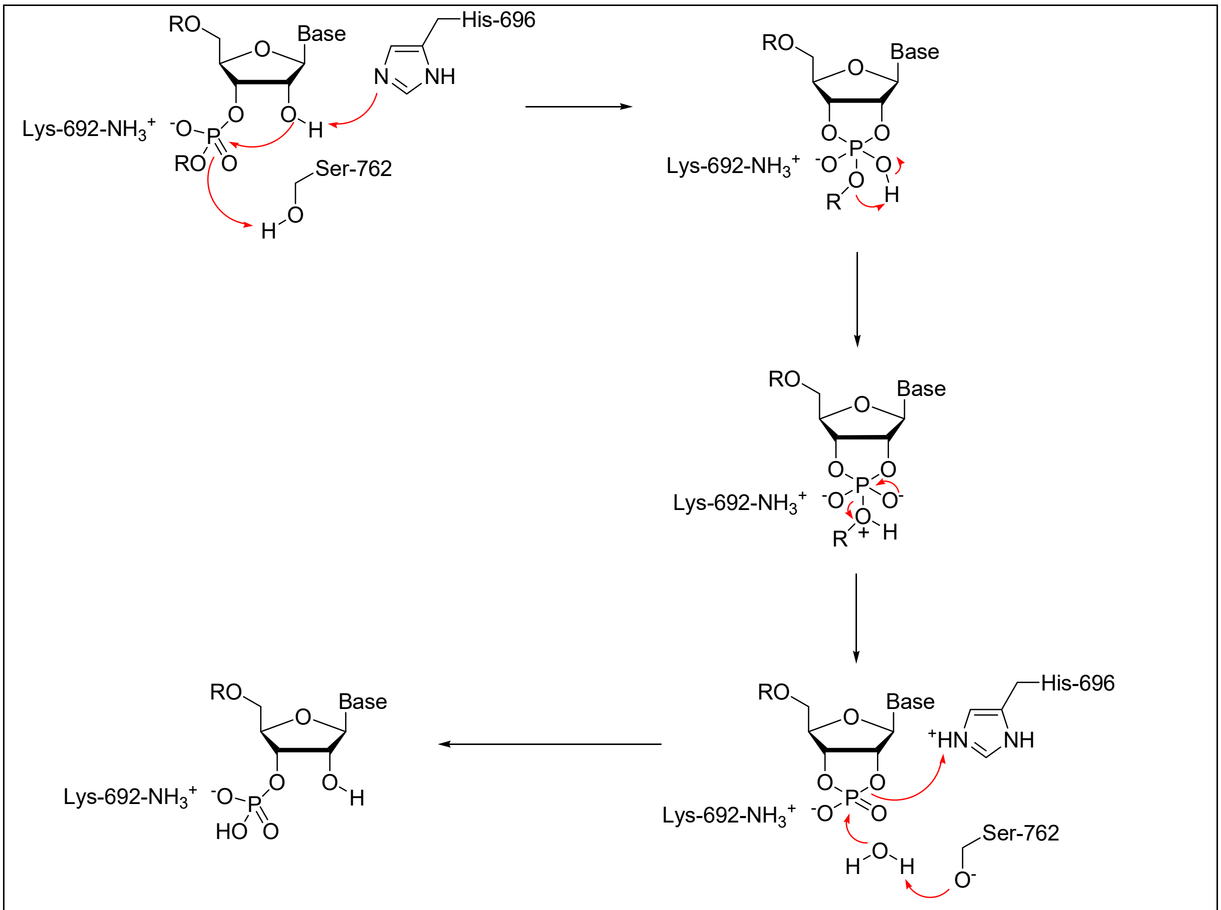

Supplementary material, Figure S5. Proposed hydrolysis mechanism of CaroS2K

Supplementary material, Table S1. Plasmids used in this study

Strain or Plasmid	Relevant characteristics	Source of reference
pES2TKD677_Q688A	Derived from pES2TKD677 with Ala substitution from position Q688	This study
pES2TKD677_K691A	Derived from pES2TKD677 with Ala substitution from position K691	This study
pES2TKD677_K692A	Derived from pES2TKD677 with Ala substitution from position K692	This study
pES2TKD677_K695A	Derived from pES2TKD677 with Ala substitution from position K695	This study
pES2TKD677_H696A	Derived from pES2TKD677 with Ala substitution from position H696	This study
pES2TKD677_Y734A	Derived from pES2TKD677 with Ala substitution from position Y734	This study
pES2TKD677_F760A	Derived from pES2TKD677 with Ala substitution from position F760	This study
pES2TKD677_S762A	Derived from pES2TKD677 with Ala substitution from position S762	This study
pES2TKD677_W764A	Derived from pES2TKD677 with Ala substitution from position Q688	This study

Supplementary material, Table S2. Primers used in this study.

S2_H696A_forT	GTATCTTAAAGCTGCCAAAGATTTTGGTATTGTTGATACC
S2_H696A_forS	TTTTGGTATTGTTGATACC
S2_H696A_revT	TCTTTGGCAGCTTTAAGATACTTTTTGTCCAGTTGCTTACG
S2_H696A_revS	TTTTTGTCCAGTTGCTTACG
S2_K698A_forT	AACATGCCGCAGATTTTGGTATTGTTGATACCAGAA
S2_K698A_forS	TATTGTTGATACCAGAA
S2_K698A_revT	CCAAAATCTGCGGCATGTTTAAGATACTTTTTGTCCAG
S2_K698A_revS	TAAGATACTTTTTGTCCAG
S2_R320A_forT	GCTGCGGAGGCAAAATTAACTGAAATCCACCAAGTGATC
S2_R320A_forS	TGAAATCCACCAAGTGATC
S2_R320A_revT	GTTAATTTTGCCTCCGCAGCGGTAATTGGATGTCTCAAC
S2_R320A_revS	GGTAATTGGATGTCTCAAC
S2_K321A_forT	GCGGAGCGAGCATTAACTGAAATCCACCAAGTGATCTC
S2_K321A_forS	AATCCACCAAGTGATCTC
S2_K321A_revT	TCAGTTAATGCTCGCTCCGCAGCGGTAATTGGATGTCTC
S2_K321A_revS	AGCGGTAATTGGATGTCTC
S2_T323A_forT	CGAAAATTAGCTGAAATCCACCAAGTGATCTCTTTTGC
S2_T323A_forS	ACCAAGTGATCTCTTTTGC
S2_T323A_revT	GGATTTCAGCTAATTTTCGCTCCGCAGCGGTAATTGG
S2_T323A_revS	CTCCGCAGCGGTAATTGG
S2_K346A_forT	CCATTTCAGGAGCAACTAAAACTGTTGCGGTTTACCAAG
S2_K346A_forS	TGTTGCGGTTTACCAAG
S2_K346A_revT	GTTTTAGTTGCTCCTGAAATGGTTGCGGCAGAGCTTTCTT
S2_K346A_revS	TTGCGGCAGAGCTTTCTT
S2_T347A_forT	TCAGGAAAAGCTAAAACTGTTGCGGTTTACCAAGAACAG
S2_T347A_forS	TGCGGTTTACCAAGAACAG
S2_T347A_revT	ACAGTTTTAGCTTTTCCTGAAATGGTTGCGGCAGAGCT
S2_T347A_revS	AATGGTTGCGGCAGAGCT
S2_R550A_forT	CTTCCCATTGCTCTGGCATTTTCTGATGAGAATGGAG
S2_R550A_forS	TTTCTGATGAGAATGGAG
S2_R550A_revT	ATGCCAGAGCAATGGGAAGATCCAGACTTCCATTTTTC
S2_R550A_revS	ATCCAGACTTCCATTTTTC
S2_T685A_forT	GATCGGTTTGCTCGTAAGCAACTGGACAAAAAGTATCT
S2_T685A_forS	AACTGGACAAAAAGTATCT
S2_T685A_revT	GCTTACGAGCAAACCGATCTGAATCCAAGGGATCGTT
S2_T685A_revS	TGAATCCAAGGGATCGTT
S2_K687A_forT	GTTTACTCGTGCGCAACTGGACAAAAAGTATCTTAAAC
S2_K687A_forS	ACAAAAAGTATCTTAAAC
S2_K687A_revT	CCAGTTGCGCACGAGTAAACCGATCTGAATCCAAGGGA
S2_K687A_revS	CGATCTGAATCCAAGGGA
S2_Q688A_forT	ACTCGTAAGGCACTGGACAAAAAGTATCTTAAACATGC
S2_Q688A_forS	AAAGTATCTTAAACATGC
S2_Q688A_revT	TTGTCCAGTGCCTTACGAGTAAACCGATCTGAATCCAAGG
S2_Q688A_revS	AAACCGATCTGAATCCAAGG

S2_K691A_forT	CAACTGGACGCAAAGTATCTTAAACATGCCAAAGATTTTGG
S2_K691A_forS	TAAACATGCCAAAGATTTTGG
S2_K691A_revT	AGATACTTTGCGTCCAGTTGCTTACGAGTAAACCGATC
S2_K691A_revS	CTTACGAGTAAACCGATC
S2_K692A_forT	CTGGACAAAGCGTATCTTAAACATGCCAAAGATTTTGGTA
S2_K692A_forS	ATGCCAAAGATTTTGGTA
S2_K692A_revT	GTTTAAGATACGCTTTGTCCAGTTGCTTACGAGTAAACCG
S2_K692A_revS	TTGCTTACGAGTAAACCG
S2_D699A_forT	CATGCCAAAGCTTTTGGTATTGTTGATACCAGAAAAAATAG
S2_D699A_forS	TTGATACCAGAAAAAATAG
S2_D699A_revT	CAATACCAAAAGCTTTGGCATGTTTAAGATACTTTTTGTC
S2_D699A_revS	TTTAAGATACTTTTTGTC
S2_R706A_forT	GTTGATACCGCAAAAAATAGTGAAACACTGACTAAATTTAG
S2_R706A_forS	AAACACTGACTAAATTTAG
S2_R706A_revT	CACTATTTTTTGCGGTATCAACAATACCAAAATCTTTGG
S2_R706A_revS	AATACCAAAATCTTTGG
S2_K707A_forT	GATACCAGAGCAAATAGTGAAACACTGACTAAATTTAG
S2_K707A_forS	AACACTGACTAAATTTAG
S2_K707A_revT	TCACTATTTGCTCTGGTATCAACAATACCAAAATCTTTGG
S2_K707A_revS	AACAATACCAAAATCTTTGG
S2_S709A_forT	CAGAAAAAATGCTGAAACACTGACTAAATTTAGAGACGC
S2_S709A_forS	TGACTAAATTTAGAGACGC
S2_S709A_revT	GTGTTTCAGCATTTTTTCTGGTATCAACAATACCAA
S2_Y734A_forT	AAGGGACAGCTCTACTTGTGAAGGATTCAAAGGTTTTC
S2_Y734A_forS	AAGGATTCAAAGGTTTTC
S2_Y734A_revT	CACAAGTAGAGCTGTCCCTTTTTCAAAAGTTTCTTTTTCC
S2_Y734A_revS	TTTCAAAAGTTTCTTTTTCC
S2_S740A_forT	GTGAAGGATGCAAAGGTTTTCTTTAACCCGAAGACGAA
S2_S740A_forS	TTTAACCCGAAGACGAA
S2_S740A_revT	GAAAACCTTTGCATCCTTCACAAGTAGATATGTCCCT
S2_S740A_revS	AAGTAGATATGTCCCT
S2_D755A_forT	GTTGTCATGGCTAAGGATAATAAATTTATTTCCGGTTGG
S2_D755A_forS	AAATTTATTTCCGGTTGG
S2_D755A_revT	ATTATCCTTAGCCATGACAACAACATTATTCGTCTTCGG
S2_D755A_revS	AACATTATTCGTCTTCGG
S2_D757A_forT	CATGGATAAGGCTAATAAATTTATTTCCGGTTGGAAGTTGG
S2_D757A_forS	ATTTCCGGTTGGAAGTTGG
S2_D757A_revT	AAATTTATTAGCCTTATCCATGACAACAACATTATTCGT
S2_D757A_revS	ACAACAACATTATTCGT
S2_F760A_forT	GATAATAAAGCTATTTCCGGTTGGAAGTTGGATGTTGATTC
S2_F760A_forS	TGGAAGTTGGATGTTGATTC
S2_F760A_revT	ACCGGAAATAGCTTTATTATCCTTATCCATGACAACAAC
S2_F760A_revS	CTTATCCATGACAACAAC
S2_S762A_forT	AAATTTATTGCCGGTTGGAAGTTGGATGTTGATTCTCAG
S2_S762A_forS	TTGGATGTTGATTCTCAG
S2_S762A_revT	CTTCCAACCGGCAATAAATTTATTATCCTTATCCATGAC
S2_S762A_revS	ATTATCCTTATCCATGAC

S2_W764A_forT	ATTTCCGGTGCGAAGTTGGATGTTGATTCTCAGCAGTAT
S2_W764A_forS	GTTGATTCTCAGCAGTAT
S2_W764A_revT	ATCCAACTTCGCACCGGAAATAAATTTATTATCCTTATCC
S2_W764A_revS	AAATTTATTATCCTTATCC
S2_D767A_forT	TGGAAGTTGGCTGTTGATTCTCAGCAGTATAAAAACTACG
S2_D767A_forS	AGCAGTATAAAAACTACG
S2_D767A_revT	GAGAATCAACAGCCAACTTCCAACCGGAAATAAATTTATTATC
S2_D767A_revS	ACCGGAAATAAATTTATTATC
